# A Novel Microbisporicin Producer Identified by Early Dereplication during Lantibiotic Screening

**DOI:** 10.1155/2015/419383

**Published:** 2015-08-04

**Authors:** Lucia Carrano, Monica Abbondi, Paola Turconi, Gianpaolo Candiani, Flavia Marinelli

**Affiliations:** ^1^Fondazione Istituto Insubrico Ricerca per la Vita (F.I.I.R.V.), Via R. Lepetit 32, 21100 Gerenzano, Italy; ^2^Dipartimento di Biotecnologie e Scienze della Vita, Università degli Studi dell'Insubria, Via J. H. Dunant 3, 21100 Varese, Italy; ^3^“The Protein Factory” Research Center, Politecnico of Milano, ICRM CNR Milano and University of Insubria, Via J. H. Dunant 3, 21100 Varese, Italy

## Abstract

With the increasing need of effective antibiotics against multi-drug resistant pathogens, lantibiotics are an attractive option of a new class of molecules. They are ribosomally synthetized and posttranslationally modified peptides possessing potent antimicrobial activity against aerobic and anaerobic Gram-positive pathogens, including those increasingly resistant to *β*-lactams and glycopeptides. Some of them (actagardine, mersacidin, planosporicin, and microbisporicin) inhibit cell wall biosynthesis in pathogens and their effect is not antagonized by vancomycin. Hereby, we apply an efficient strategy for lantibiotic screening to 240 members of a newly described genus of filamentous actinomycetes, named *Actinoallomurus*, that is considered a yet-poorly-exploited promising source for novel bioactive metabolites. By combining antimicrobial differential assay against *Staphylococcus aureus* and its L-form (also in the presence of a *β*-lactamase cocktail or Ac-Lys-D-alanyl-D-alanine tripeptide), with LC-UV-MS dereplication coupled with bioautography, a novel producer of the potent microbisporicin complex was rapidly identified. Under the commercial name of NAI-107, it is currently in late preclinical phase for the treatment of multi-drug resistant Gram-positive pathogens. To our knowledge, this is the first report on a lantibiotic produced by an *Actinoallomurus* sp. and on a microbisporicin producer not belonging to the *Microbispora* genus.

## 1. Introduction

Lantibiotics, the abbreviation for “lanthionine containing antibiotics,” are a class of ribosomally synthetized and posttranslationally modified peptides produced by and active versus Gram-positive bacteria [1, 2]. They are characterized by the thioether-containing linkages lanthionine (Lan) and/or methyllanthionine (MeLan), originating by the dehydration of Ser/Thr residues in a precursor peptide followed by intramolecular addition of Cys to the dehydrated residues. Nisin, the best characterized lantibiotic, has been used as a food preservative to combat food-borne pathogens for more than forty years without the development of widespread antibiotic resistance [3]. As such, lantibiotics are a promising group of natural products to battle the continuous rise of antibiotic resistance [4]. Some of them like actagardine [5], mersacidin [6], planosporicin [7], and microbisporicin [8] possess potent antimicrobial activity against aerobic and anaerobic Gram-positive pathogens, including those increasingly resistant to *β*-lactams and glycopeptides [9]. They inhibit cell wall biosynthesis [10] without showing cross-resistance with vancomycin [11]. Furthermore, lantibiotics have been shown to have promising efficacy and pharmacokinetics in animal models [12, 13].

The renewed interest for this class of specialized microbial metabolites has prompted in the last decade the search of novel lantibiotics following different approaches: (i) by chemical modification of known molecules [14]; (ii) by gene site-directed mutagenesis and expression of lantibiotics' variants in heterologous hosts [15–17]; (iii) by screening untapped microbial diversity for novel scaffolds [7, 8, 18]. It is widely recognized that the success of the last approach depends mostly on the novelty of the microbial sources and on the selectivity of the screening strategy [19, 20]. Presently, after decades of massive natural product screening, one of the limiting hindrance is the reisolation of already discovered bioactive molecules [21]. Since structure elucidation of a natural product purified from a complex matrix such as microbial extract is a demanding step, early identification of known or undesirable compounds, hereby indicated as dereplication, is a key activity in microbial natural product screening, saving resources and speeding up the discovery process of novel drugs [19–22].

In this work, we combine a robust and selective lantibiotic screening strategy applied to a newly described genus of filamentous actinomycetes named* Actinoallomurus* [23] with an early procedure of dereplication. Recent papers claim that* Actinoallomurus* is a good source of novel bioactive metabolites [24, 25], but to our knowledge it has not been yet exploited for the production of lantibiotics.

## 2. Materials and Methods

### 2.1. Bacterial Strains

*Staphylococcus aureus* 209 ATCC 6538P (L100) were purchased from the American Type Culture Collection (ATCC; Manassas VA). L-form cells (L3751) were prepared from L100 by exposure to 100 U of penicillin in Enterococcal Brain Heart Infusion/S (EBH/S) supplemented with 5% NaCl, 5% sucrose, and 10% horse serum as previously described [5, 26]. L-forms were then cultured on similarly supplemented brain heart infusion agar containing no antibiotic.* S. aureus* Smith ATCC19636 (L819),* Streptococcus pyogenes* C203 ATCC12384 (L49), and other clinical isolates (*S. aureus* L1400,* Enterococcus faecalis* L559,* Enterococcus faecalis* Van A L560,* Escherichia coli* SKF12140 L47, and* Candida albicans* SKF2270 L145) were maintained in the Fondazione Istituto Insubrico Ricerca per la Vita (F.I.I.R.V.) culture collection (L collection) at Gerenzano, Italy.

### 2.2. Media and Culture Conditions

*Actinoallomurus* spp. were isolated from different soil sources with the following method: 250 mg finely ground and dried soil (100°C for 60 min) was poured onto agar plates of HSA5.5 medium (in g/L: humic acid, 2 previously dissolved in 10 mL 0.2 NaOH aqueous solution; FeSO_4_·7H_2_O, 0.001; MnCl_2_·4H_2_O, 0.001; ZnSO_4_·7H_2_O, 0.001; NiSO_4_·6H_2_O, 0.001; MES, 2; agar, 20; add 1 mL CMM vitamin solution containing 25 *μ*g thiamin hydrochloride, 250 *μ*g calcium pantothenate, 250 *μ*g nicotinic acid, 500 *μ*g mg biotin, 1,25 mg riboflavin, 6 *μ*g vitamin B_12_, 25 *μ*g* p*-aminobenzoic acid, 500 *μ*g folic acid, and 500 *μ*g pyridoxal hydrochloride; pH adjusted to 5.5 before sterilization). All the medium components were purchased from Sigma-Aldrich, unless otherwise stated. Isolation plates were incubated at 50°C for 24 h and then at 28°C for more than four weeks. Pure colonies were picked up, checked at the microscope, and then maintained at 28°C on pH 5.5 ISP3 agar plates. Morphology was observed at the stereoscope (Zeiss) and at the light microscope (model ULWD-CDPlan; Olympus) fitted with a 3CCD camera (Sony). For liquid cultures, a loopful of mycelium was scrapped off and transferred in a 80 mL baffled Erlenmeyer flask containing 15 mL of AF5 (g/L: dextrose, 20; yeast extract, 2; soybean meal, 8; NaCl, 1; and MES, 10; pH adjusted to 5.5 before sterilization) or M85.5 (g/L: dextrose, 10; yeast extract, 2; beef extract 2; starch, 20; casein hydrolysate, 2; and MES, 20; pH adjusted to 5.5 before sterilization). Unless otherwise stated, all fermentation medium components were from Constantino, Arese, Italy. After six days, 10% (v/v) of the culture was transferred into 500 mL flasks containing 100 mL of AF5 or M85.5. Flasks were incubated for 16–18 days at 28°C on a rotary shaker at 200 rpm. After centrifugation at 3000 rpm for 15 min, broths (10 mL) were extracted by adding 2.3% (v/v) polystyrenic resin HP-20 (Mitsubishi Chemical Co.) and eluting it batchwise with 5 mL pure methanol (screening broth extracts). For the preparation of a partially purified fraction (crude extract), the strain was grown as reported above in 1000 mL flasks containing 350 mL AF5 medium. Approximately, 300 mL broth was loaded on HP-20 resin (7.5 mL) that was eluted stepwise by increasing the organic phase percentage: first by 30 mL of methanol : water 2 : 3 (v/v), then by 30 mL methanol : water 4 : 1 (v/v), and finally by 30 mL methanol : isopropanol 9 : 1 (v/v). The last eluted fraction was concentrated to dryness in rotavapor. Preparative chromatography was followed by UV spectroscopy and bioactivity (see below). Mycelium extracts were prepared by directly adding 2 mL ethanol per gram wet mycelium; samples were shaken at 200 rpm for 2 h. The organic phases were finally concentrated to dryness under a N_2_ flow in a Turbo-Vap unit and stored at −10°C.

### 2.3. Lantibiotic Screening Differential Assay

Broth and mycelium screening extracts from the F.I.I.R.V. collection of* Actinoallomurus* strains isolated according as above were screened in liquid microplate assays for their antimicrobial activity on* S. aureus* 209 ATCC 6538P (L100) and to its L-form cells (L3751), as described in detail in [7]. In brief,* S. aureus* 209 ATCC 6538P (L100) and its L-form cells (L3751) were maintained at −80°C in Nutrient Broth (Difco) to which 20% (v/v) glycerol was added. EBH/S supplemented with 5% (v/v) horse serum was used as medium. For the wild-type inoculum, 10 *μ*L of extracts previously dissolved in DMSO : H_2_O 1 : 9 (v/v) were added to 1 × 10^5^ CFU/mL in 90 *μ*L of culture broth. For L-form cells, aliquots of liquid cultures grown overnight in EBHI/S to O.D._620 nm_ = 0.2 were used as inoculum. Incubation time was 24 h at 35°C in air, and then growth inhibition was measured at O.D._620 nm_. Reference actagardine, planosporicin, microbisporicin, mersacidin, and nisin standards were used [7, 8] and MIC levels were determined by broth microdilution assay as recommended by the National Committee for Clinical Laboratory Standards [27]. To identify *β*-lactam producers, antimicrobial activity versus* S. aureus* 209 ATCC 6538P (L100) was measured in a liquid microplate assay after adding the following cocktail of *β*-lactamases: Penicillase Type I from* Bacillus cereus* (Sigma P0389), 0.001 U/mL; Penicillase Type II from* Bacillus cereus* (Sigma P6018), 0.002 U/mL; Penicillase type III from* Enterobacter cloacae* (Sigma P4399), 0.0025 U/mL; and Penicillase type IV from* Enterobacter cloacae* (Sigma P4524), 0.5 U/mL. To identify glycopeptide producers, antimicrobial activity versus* S. aureus* 209 ATCC 6538P (L100) was measured in a liquid microplate assay after adding 2 mg/mL of Ac-Lys-D-alanyl-D-alanine (Chem-Impex International Inc., IL).

### 2.4. LC-UV-MS and MS/MS Analyses

LC-MS and MS/MS experiments were performed in a ThermoQuest Finnigan LCQ Advantage mass detector equipped with an ESI interface and Thermo Finnigan Surveyor MS pump, photo diode array detector (PDA) (UV6000; Thermo Finnigan), and an autosampler. The Thermo Surveyor HPLC instrument was equipped with a Symmetry C18 (5 *μ*m, 4,6 × 250 mm Waters Chromathography) column. Analyses were performed at 1 mL/min flow rate according to a multistep linear gradient using phase B (acetonitrile) in phase A (acetonitrile: 10 mM ammonium formiate pH 4.5 buffer, 5 : 95 v/v). The column was equilibrated in 20% phase B; after 1 min in these conditions, the concentration of phase B increased up to 90% in 31 min, followed by further 4 min at 90% phase B. Full UV-visible spectra of the eluted molecules, 200–600 nm range, were detected by PDA. MS spectra were obtained by electrospray ionization, both in positive and in negative mode. MS/MS were performed on the same apparatus by changing ionization energy both in positive and negative mode. The ThermoQuest Finnigan LCQ Advantage mass detector was previously tuned and calibrated in electrospray mode in the following conditions: Spray Voltage: 4.5 kV; Capillary temperature: 220°C; Capillary Voltage: 3 V. LC/MS/MS were performed on the same apparatus in dependent scan mode, mass range 900–1200, default charge state 2, and enabling charge screening, using a normalized collision energy (CID) of 30 ev, Act Q 0.250 Act TIME (ms) 30.

For bioautography, fractions (1 mL, eluting at 1 mL/min) from the HPLC column were collected, dried, and resuspended in 100 *μ*L aqueous solution at 10% (v/v) DMSO. 10 *μ*L were tested for antimicrobial activity. UV and mass spectra of molecules present in the active fractions were compared with those collected in the ABL database, which contains data on approximately 30,000 microbial metabolites collected from literature and patents since 1950 [20, 28], and in the commercially available Antibase (http://wwwuser.gwdg.de/~hlaatsc/antibase.htm).

### 2.5. Antimicrobial Activity

Antimicrobial activity was determined by broth microdilution assay according to standard guidelines [27]. The growth media utilized to determine the MIC were cation-adjusted Difco Mueller Hinton Broth (MHB) for* Staphylococci*,* Enterococci*, and* E. coli*, Todd Hewitt Broth (THB) for* Streptococci*, and RPMI-1640 medium (RPMI) for* C. albicans*. Typically, a twofold serial dilution of the test compound was performed in a sterile 96-well microplate inoculated with 10^4^ CFU/mL of the test strain in the appropriate medium. The microplate was then incubated for 18–24 h at 35°C. The MIC was determined by visual examination of the microplates with the aid of a magnifying mirror as the lowest concentration of antibiotic that showed no visible sign of microbial growth.

### 2.6. 16S rRNA Gene Sequencing

Genomic DNA was extracted with the GenElute Bacterial Genomic DNA kit (Sigma-Aldrich) by colony picking; PCR-mediated amplification of the 16S rRNA gene, purification of the PCR products and sequencing were carried out as previously described [29]. Alignments of 16S rRNA gene sequences were conducted with BLASTN (http://www.ncbi.nlm.nih.gov/blast/). For the construction of the phylogenetic tree, selected sequences were aligned with Clustal-Omega (from the EMBL-EBI site) and analyzed with BioEdit [30]. Distance matrices were calculated with MEGA5.2, using the Maximum Likelihood method implemented in the program and the method of Jukes and Cantor. Trees were inferred using the Nearest-Neighbor-Interchange (NNI) heuristic method and making the initial tree with both Neighbour Joining and BioNJ, and selecting the superior tree (all methods are included in the MEGA package). All analyses were performed on a bootstrapped data set containing 500 replicates.

## 3. Results and Discussion

### 3.1. Lantibiotic Screening of* Actinoallomurus* spp. 

880 extracts were obtained from broth and mycelium of 240* Actinoallomurus* spp. (from the F.I.I.R.V. collection) isolated as described in Section 2, after six days of growth in fermentation media AF5 and M85.5. Primary screening was based on the differential activity assay versus* S. aureus* and its L-form. L-forms are protoplast-type cells derived from* S. aureus* that are able to replicate in appropriate osmotic conditions despite the lack of a functional cell wall [5, 7, 26]. As previously shown in [7], L-forms are equally or more sensitive than parental cells to those antibiotics acting on molecular targets other than cell wall biosynthesis. They are indeed resistant to peptidoglycan synthesis inhibitors. Extracts from 67 strains were equally active on* S. aureus* and its L-form, whereas only 2 strains gave a significant level of differential activity: their MICs versus L-form cells were at least eightfold higher than those against the whole cells. Secondary selection was based on whether antimicrobial activity against* S. aureus* could be reversed by a *β*-lactamase cocktail or by adding Ac-Lys-D-alanyl-D-alanine tripeptide, which mimics the glycopeptide cell target. This step was introduced to eliminate PG inhibitors belonging to the known classes of *β*-lactams and glycopeptides. Only one strain (named F31/11) passed the secondary selection: its activity versus* S. aureus* was not abolished by adding either the *β*-lactamase cocktail or the Ac-Lys-D-alanyl-D-alanine tripeptide. F31/11 antimicrobial activity was reconfirmed upon its repeated fermentation, and it was found to be excreted into the medium (Table 1) as well as being associated to the mycelium (data not shown). Both extracts were found active against clinical isolates representative of Gram-positive pathogens, including one methicillin resistant* S. aureus* (MRSA) and one vancomycin-resistant* E. faecalis* (VanA). The Gram-negative* E. coli* was insensitive and, consistent with the mode of action of bacterial cell wall inhibitors, no activity was observed against* S. aureus* L-form (L3751) and the eukaryote* C. albicans*.

### 3.2. Antimicrobial Activity of F31/11

The pattern of antimicrobial activity of F31/11 extract shown in Table 1 matches with the one expected for a potent lantibiotic. To confirm this, we prepared an enriched crude extract as described in Section 2 by partition chromatography from F31/11 broth, which was tested in parallel with standard samples of lantibiotics (actagardine, planosporicin, microbisporicin, mersacidin, and nisin). Data reported in Table 2 confirm the antimicrobial potency of the unknown antibiotic produced by F31/11.

### 3.3. LC-UV-MS Coupled with Bioautography

**Figure 1:**
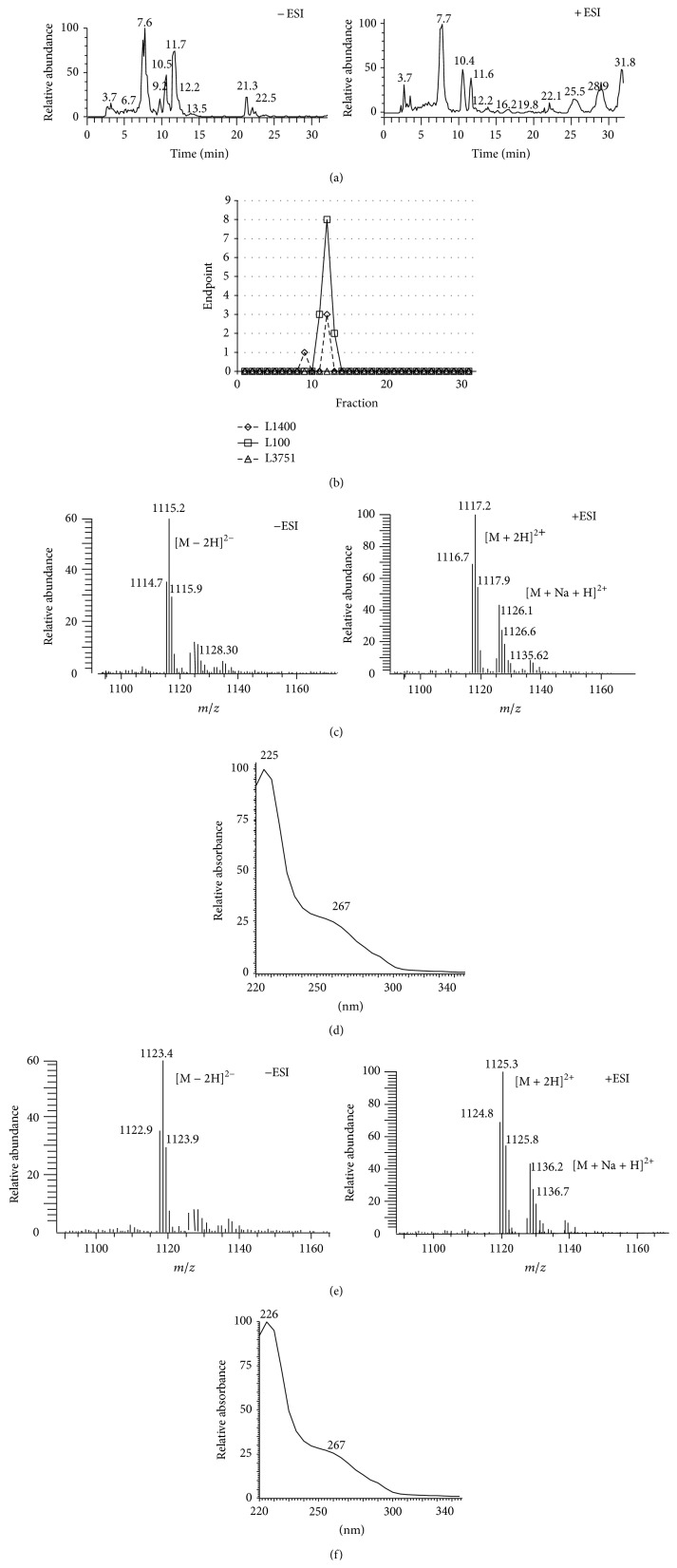
MS-HPLC profiles of the F31/11 broth screening extract: (a) MS trace in negative and positive mode; (b) bioautography: each HPLC fraction was tested versus* S. aureus* MRSA L1400, MSSA L100, and L-form L3751 in dose dilution; (c) MS spectrum of the peak eluting at 11.7 min in negative and positive mode; (d) UV spectrum of the peak eluting at 11.7 min; (e) MS spectrum of the peak eluting at 12.2 min in negative and positive mode; (f) UV spectrum of the peak eluting at 12.2 min. In UV spectra, the *λ* values of the maximum and of the shoulder are indicated.

UV and MS spectra were simultaneously collected during HPLC chromatography fractionation and each chromatographic fraction was in parallel tested for antimicrobial activity versus* S. aureus*, its L-form and versus a MRSA clinical isolate, conducting the so called bioautography (Figure 1).  Figure 1(a) shows the presence of many compounds in the MS-HPLC profile by electrospray ionization, both in positive and in negative mode, within the crude extract from F31/11. Fractionation coupled with the activity profile shown in Figure 1(b) indicates a major peak eluting at* ca*. 11.7 min (−ESI) and 11.6 (+ESI), which corresponds to the putative lantibiotic, which inhibits the microbial growth of* S. aureus*, but not its L-form. Base peak ion extraction pointed out that the molecule eluting at 11.7 min has* m/z* of 1115.2 in negative mode (−ESI) and of 1117.2 in positive mode (+ESI). MS spectrum (Figure 1(c)) shows that the lowest molecular weight signals correspond to double charged species, more exactly to the double-charged ion [M + 2H]^2+^ at* m/z* of 1117.2, [M + Na + H]^2+^ at* m/z* of 1126.1, and [M − 2H]^2−^ at* m/z* 1115.2, suggesting a molecular weight of 2230 Da. As shown in Figure 1S in Supplementary Material available online at http://dx.doi.org/10.1155/2015/419383, the full scan mass spectrum range of 1000–3000 mass units value of this peak shows the presence of the signal corresponding to the single-charged ion [M + H]^+^ at* m/z* of 2231.2. The UV spectrum shows two shoulders at 225 and 267 nm (Figure 1(d)).

The bioautography of the mycelium extract led to the identification of the same molecular species eluting at 11.7 min and highlighted the presence of a second peak eluting at 12.2 min. This peak was also present (but in lower amount) in the LC/MS profile from the broth extract (Figure 1(a)). This last peak shows a similar UV profile as the one at 11.7 min, showing two shoulders at 226 and 267 nm (Figure 1(f)). It is characterized by a double-charged ion [M + 2H]^2+^ at* m/z* of 1125.3, a double-charged ion [M + Na + H]^2+^ at* m/z* 1136.2 in positive current ion, and a signal corresponding to the double-charged ion [M − 2H]^2−^ at* m/z* of 1123.4 in the negative mode (Figure 1(e)). As shown in Figure 1S in Supplementary Material, the full scan mass spectrum range of 1000–3000 mass units value of this peak shows the presence of the signal corresponding to the single-charged ion [M + H]^+^ at* m/z* of 2247.2.

**Figure 2:**
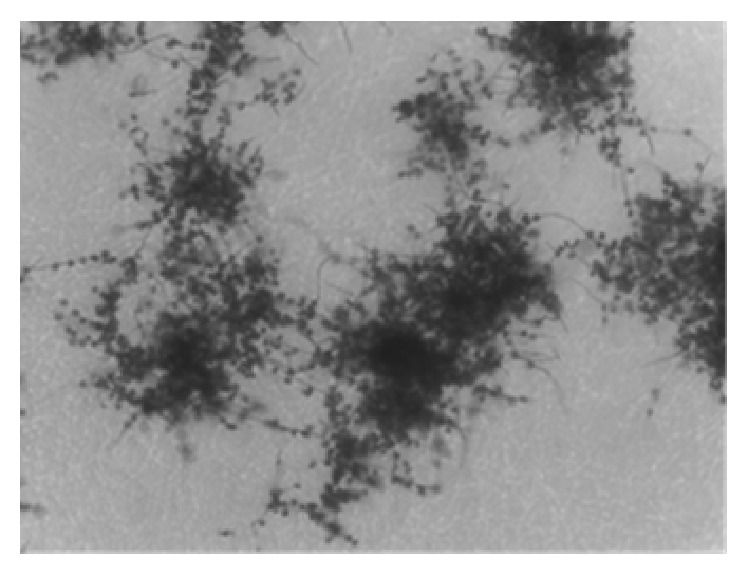
Morphology of F31/11 observed at the light microscope (model ULWD-CDPlan; Olympus, with 40x magnification).

To gain further information on the structure of the two active compounds eluting at 11.7 and 12.2 min, we investigated them by further runs of LC/MS/MS: the signal corresponding to* m/z* of 1117.2 originated an intense peak at* m/z* of 1099.54, while in the same conditions the signal at* m/z* 1125.3 originated an intense signal at* m/z* 1107.6 (Figure 2S, Supplementary Material). These MS/MS spectra indicate that the parent ions did not easily fragmented by the collision energy of 30 ev used in this study, and this is probably due to the typical lantibiotic structure, where the presence of (Me)Lan bridges requires higher collision energy for generating fragments.

When these UV and MS data were matched with the information stored in databases ABL [20, 28] and Antibase, the compound eluting at 11.7 min present in the broth crude extract (and to a lesser extent in the mycelium) was identified as the A2 congener of microbisporicin, while the compound eluting at 12.2 from the mycelium extract (and to a lesser extent from the broth extract) was identified as the A1 congener of microbisporicin. It is important to note that A1 and A2 congeners of microbisporicin differ for the presence of dihydroxy- or hydroxyl-proline in the aminoacidic sequence, equivalent to a difference of one oxygen in the molecular formula, respectively, C_94_H_127_ClN_26_O_27_S_5_ and C_94_H_127_ClN_26_O_26_S_5_. Thus, the difference observed through LC/MS/MS between F31/11 active component eluting at 11.7 and F31/11 active component eluting at 12.2 min (Figure 2S in the Supplementary Material) could be explained by the presence of an additional oxygen on proline. Figure 3S in Supplementary Material confirms that when the A1 congener of microbisporicin was analyzed by LC/MS/MS in parallel with the compound eluting at 12.2 min, the two molecules originate the same fragmentation signals, reported in Figure 3S of the Supplementary Material. The identification of the two active components produced by F31/11 as the A1 and A2 congeners of microbisporicin was then further confirmed by LC-UV-MS analyses of F31/11 extracts in parallel with standards of actagardine, planosporicin, and microbisporicin (Table 3).

Microbisporicin is the most potent antibacterial among the known lantibiotics [8]; under the commercial name of NAI-107, it is currently in late pre-clinical phase for the treatment of multi-drug resistant Gram-positive pathogens [12, 13]. So far, two actinomycetes both belonging to the* Microbispora* genus have been reported to produce a different complex of microbisporicin congeners:* Microbispora* sp. 107981 mostly produces A1 and A2 congeners differing by the presence of dihydroxy- or hydroxyl-proline at position 14 in the 24 amino acid long scaffold [8]. Other minor congeners produced by the same strain have been recently identified, carrying possible permutations on the tryptophan residue at position 4 (no modification or chlorination) and on the proline at position 14 (no modification or mono- or di-hydroxylation) [31].* Microbispora corallina* NRRL 30420 produces mostly 1768*β* (no modification on proline at position 14) and 1768*α* (not chlorination on tryptophan at position 4 and no modification on proline at position 14) and lower amount of A1 and A2 [31–33]. We cannot exclude that other minor components could be produced by F31/11 strain, but the data reported in Table 3 indicate that, in the cultivation conditions so far used, it coproduces A2 and A1 congeners, preferentially accumulating A2 into the broth. We can add that the isotopic profile of the mass spectrum of F31/11 active peaks confirms the presence of chlorine in the molecule (data not shown).

### 3.4. Characterization of the F31/11 Producer Strain

Isolates belonging to the F.I.I.R.V. microbial collection were initially attributed to the* Actinoallomurus* genus mainly on the basis of their morphological and physiological features and by 16S rRNA gene sequencing [23, 24]. Typically,* Actinoallomurus* sp. F31/11 grows well at 30–37°C on ISP3 agar acidified to pH 5.5–6.0 with HCl. It forms typical chains of looped spores (Figure 2); the substrate mycelium is convolute and the mass colour of the substrate mycelium is cream. Good production of white-grey aerial mycelium was observed after 15 days of incubation. No soluble pigments are produced.

**Figure 3:**
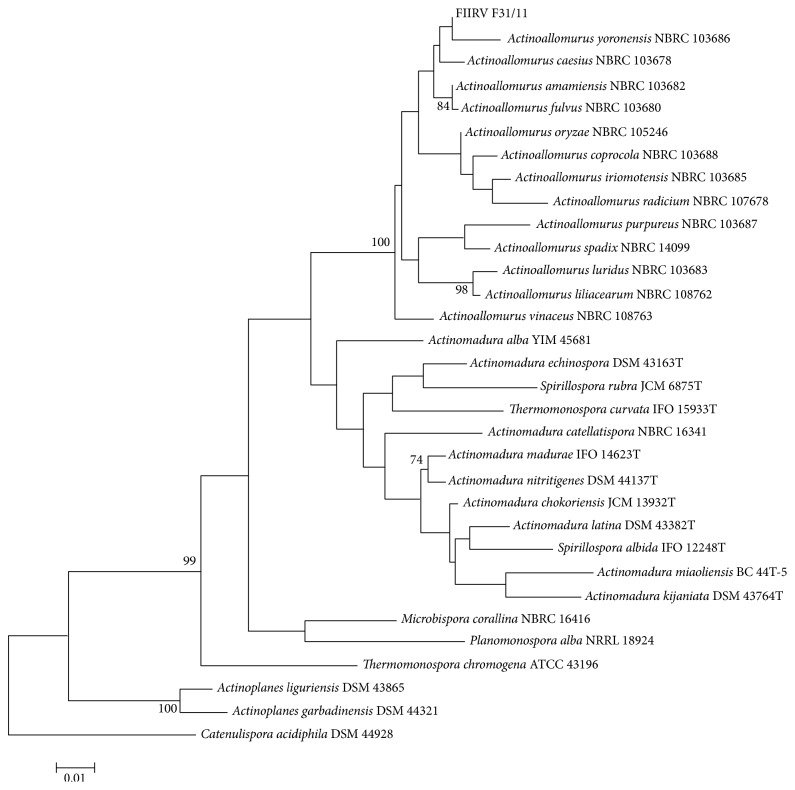
Phylogenetic tree derived from the 16S rRNA gene sequences of* Actinoallomurus* species and related actinomycetes belonging to the* Thermomonosporaceae* family. Sequences from actagardine, planosporicin, and microbisporicin actinomycete producers were also included. For the construction of the phylogenetic tree, selected sequences were aligned with Clustal-Omega (from the EMBL-EBI site) and analyzed with BioEdit [30]. Distance matrices were calculated with MEGA5.2, using the Maximum Likelihood method implemented in the program and the method of Jukes and Cantor. Trees were inferred using the Nearest-Neighbor-Interchange (NNI) heuristic method and making the initial tree with both Neighbour Joining and BioNJ, and selecting the superior tree (all methods are included in the MEGA package). All analyses were performed on a bootstrapped data set containing 500 replicates.

The taxonomical affiliation of strain F31/11 to the genus* Actinoallomurus* was confirmed by pairwise comparison of its almost complete 16S rRNA gene (1400 bp) with those of already described members of the* Actinoallomurus* genus (Figure 3) [23]. F31/11 16S rRNA sequence showed an identity of 99% with* Actinoallomurus yoronensis, Actinoallomurus fulvus, Actinoallomurus caesius*, and* Actinoallomurus amamiensis*. This identity value is indeed lower than 99.5%, which is considered the threshold for distinguishing different phylotypes; thus, F31/11 might be considered a novel species. The phylogenetic tree shown in Figure 3 clearly indicates that F31/11 with other* Actinoallomurus* spp. form a distinct clade within the* Thermomonosporaceae* family and that F31/11 is quite distant from the microbisporicin producer* Microbispora corallina* (*Streptosporangiaceae* family) as well as from other lantibiotic producing actinomycetes such as* Planomonospora alba* (*Streptosporangiaceae* family) that produces planosporicin [7, 34] and from* Actinoplanes garbadinensis* and* Actinoplanes liguriensis* (*Micromonosporaceae* family) that produce actagardine [35].

## 4. Conclusions

As far as we know, this is the first report on a lantibiotic produced by an* Actinoallomurus* sp. and on a microbisporicin producer not belonging to the* Microbispora* genus. Unrelated compounds belonging to different chemical classes (benanomicin, coumermycin, N-butylbenzensulphonamide, and halogenated spirotetronates) have been recently discovered as products of* Actinoallomurus* spp. [24, 25], confirming that this novel genus represents a promising source for discovering novel bioactive metabolites when targeted with selective and efficient screening strategies. While most lantibiotics have been previously isolated and characterized from different genera of* Firmicutes*, recent investigations [7, 8, 18, 31–34] indicate that uncommon actinomycetes (non-streptomyces actinomycetes) can effectively contribute to the discovery of novel and useful lantibiotics. The case reported here suggests that same lantibiotic scaffolds may be produced by diverse families of actinomycetes. Thus, coupling an intelligent biological-activity guided screening with an early efficient dereplication approach avoid spending time in labour intensive procedure of purification and structural elucidation of already known metabolites. As recently reviewed in [36], implementing efficient, early LC-MS dereplication platform to identify known compounds in natural product databases containing their spectra, is nowadays considered a strategic step in natural product discovery. Further investigations will be devoted to understanding the potential of* Actinoallomurus* spp. as specialized metabolite producers.

## Supplementary Material

Supplementary Material reports additional MS and MS/MS data on F31/11 active fractions.

## Figures and Tables

**Table 1:** Antimicrobial activity of the screening extract from F31/11 broth measured as an endpoint in microdilution method, that is, the highest dilution that inhibits 80% of test strain growth.

Microorganism	Medium	Active dilution
L100* S. aureus *ATCC 6538P	EBH/S	>1 : 64
L3751 *S. aureus *L-form	EBH/S	<1 : 4
L100 *S. aureus *ATCC 6538P	EBH/S + *β*-lactamase cocktail	1 : 64
L100 *S. aureus *ATCC 6538P	EBH/S + Ac-Lys-D-Ala-D-Ala	1 : 64
L1400 *S. aureus *MRSA	MHB	1 : 64
L49 *S. pyogenes *	THB	>1 : 64
L559 *E. faecalis *	MHB	1 : 8
L560 *E. faecalis *Van A	MHB	1 : 16
L47 *E. coli *	MHB	<1 : 4
L145 *C. albicans *	RPMI	<1 : 4

**Table 2:** Antimicrobial activity of F31/11 crude extract in comparison to planosporicin, actagardine, microbisporicin, mersacidin, and nisin standards. MICs were determined by broth microdilution assay [27].

Strain	MIC (mg/L)					
	Planosporicin	Actagardine	Microbisporicin	Mersacidin	Nisin	F31/11
L100 *S. aureus* ATCC6538P	2	32	≤0.13	4	0.5	4
L3751 *S. aureus *L-form	>128	>128	>128	64	16	>128
L1400 *S. aureus *MRSA	16	16	≤0.13	8	2	8
L49 *S. pyogenes *	<1	2	<1	n.d	n.d	1
L47 *E. coli *	>128	>128	>128	n.d	>128	>128
L145 *C. albicans *	>128	>128	>128	n.d	>128	>128

**Table 3:** Retention time and typical UV and mass signals of actagardine and planosporicin and of major microbisporicin congeners in the LC-UV-MS system described in Section 2. Mass signals are reported in Dalton. *λ*_1_ and *λ*_2_ signals indicate, respectively, lambda (max) and lambda (shoulder).

ANTIBIOTIC	M	r.t. (min)	[M + 2H]^2+^	[M − 2H]^2−^	[M + H]^+^	UV nm (*λ*_1_ and *λ*_2_)
Actagardine	1889	10.6	944.5	943.5	1890	227, 282
Microbisporicin A1	2246	12.2	1125.3	1123.4	2247	226, 267
Microbisporicin A2	2230	11.7	1117.2	1115.3	2231	225, 267
Microbisporicin 1768*α*	2214	12.8	1108.5	—	2215	223, 270
Microbisporicin 1768*β*	2180	9.6	1091	—	2181	223, 270
Planosporicin	2196	8.7	1099.7	1097.7	2197	225, 279, 288
F31/11 broth extract	2230	11.7	1117.2	1115.2	2231	225, 267
F31/11 mycelium extract	2246	12.2	1125.3	1123.4	2247	226, 267
