# Supplementary material for: A Novel Microbisporicin Producer Identified by Early Dereplication during Lantibiotic Screening
Source: Biomed Res Int. 2015 Aug 4;2015:419383. doi: 10.1155/2015/419383 (PMC4539421; doi:10.1155/2015/419383)

**Supplemental Material**

**Figure S1.** MS spectra of F31/11: full scan low resolution spectrum of active components eluting at 1.7 (upper panel) and 12.2 min (lower panel).


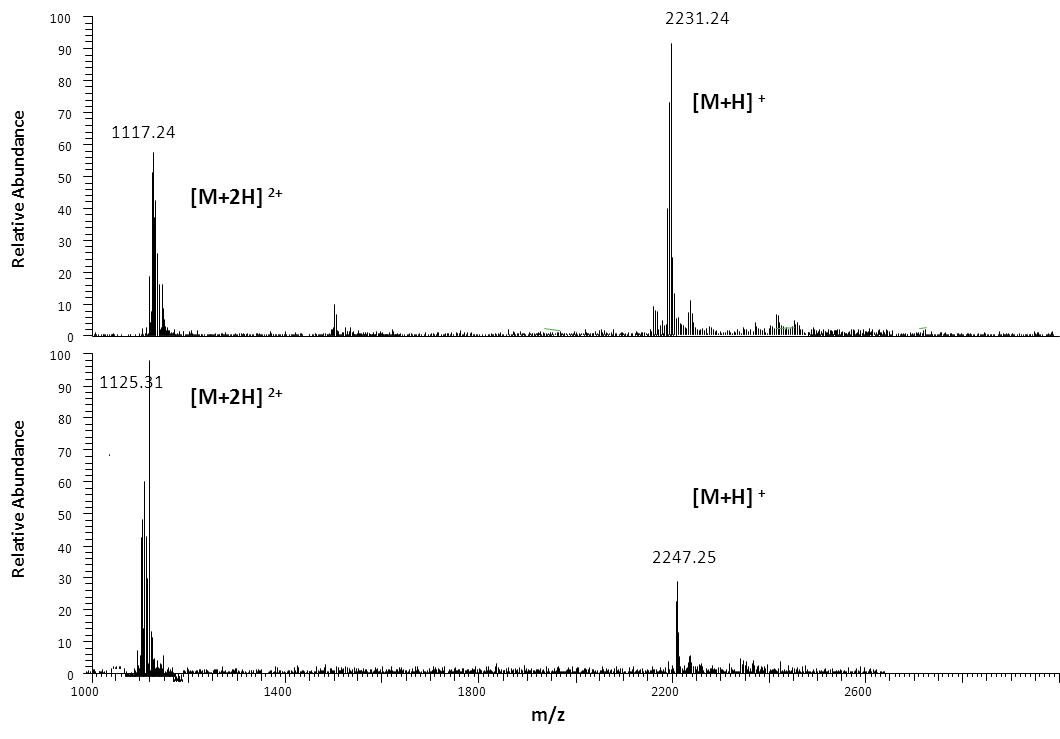


**Figure S2**. The major fragments from MS/MS of the F31/11 active components eluting at 11.7 min (upper panel) and 12.2 min (lower panel). Spectra were obtained by the collision energy of 30ev.


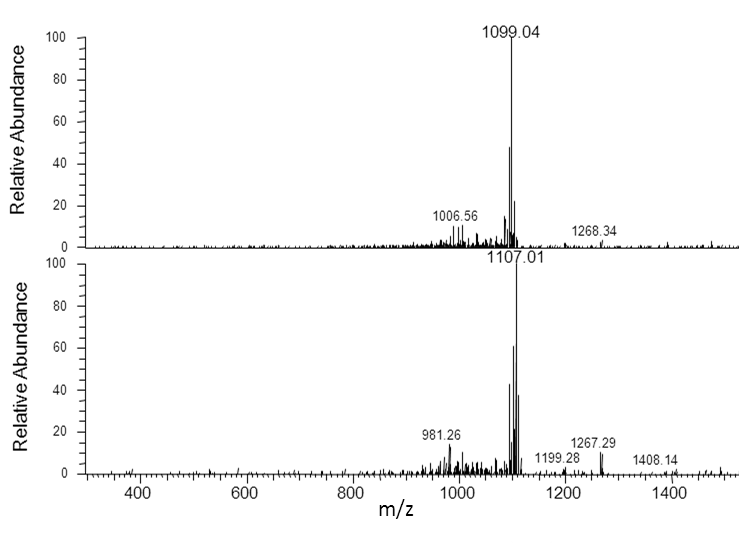


**Figure S3**. The major fragments from MS/MS of standard Microbisporicin A1 produced by *Microbispora corallina* (upper panel) and from the F31/11 active component eluting at 12.2 min (lower panel). Spectra were obtained by the collision energy of 30ev.


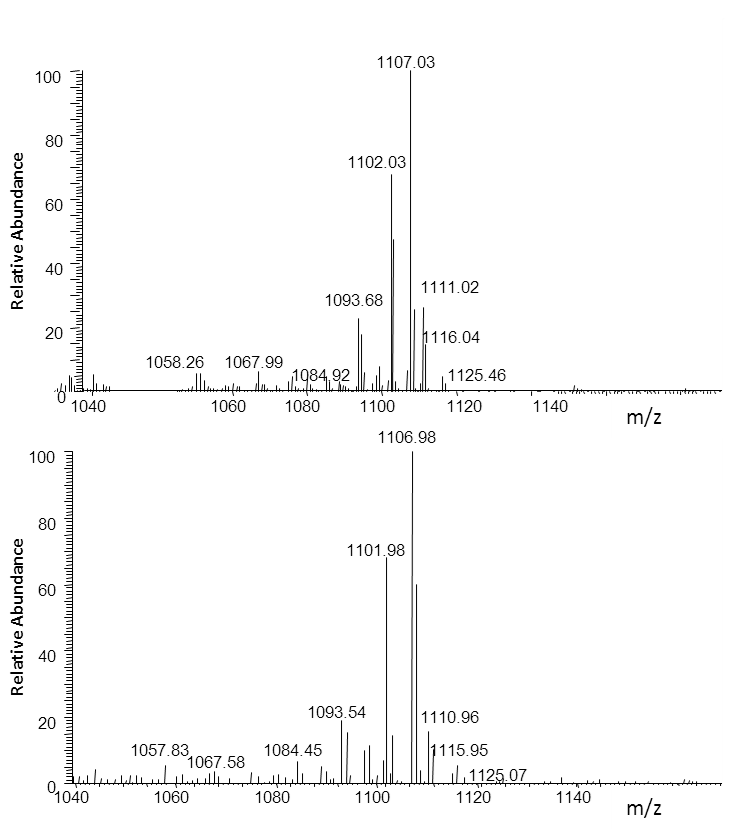

Supplement: Supplementary file 1 — Supplementary Material reports additional MS and MS/MS data on F31/11 active fractions. [file 419383.f1.docx]
